# The effect of citrate dialysate on intradialytic heparin dose in haemodialysis patients: study design of a randomised controlled trial

**DOI:** 10.1186/s12882-015-0144-z

**Published:** 2015-08-25

**Authors:** Davina J. Tai, Kelvin Leung, Pietro Ravani, Robert R. Quinn, Nairne Scott-Douglas, Jennifer M. MacRae, For the Alberta Kidney Disease Network

**Affiliations:** Department of Medicine, University of Saskatchewan, Saskatoon, Saskatchewan Canada; Department of Medicine, University of Calgary, Calgary, Alberta Canada; Department of Community Health Sciences, University of Calgary, Calgary, Alberta Canada

**Keywords:** Citrate dialysate, Hemodialysis, Haemodialysis, Unfractionated heparin, Anticoagulation, Randomised controlled trial

## Abstract

**Background:**

Unfractionated heparin is the most common anticoagulant used in haemodialysis (HD), although it has many potential adverse effects. Citrate dialysate (CD) has an anticoagulant effect which may allow reduction in cumulative heparin dose (CHD) compared to standard acetate dialysate (AD).

**Methods:**

This double-blinded, randomised, cross-over trial of chronic haemodialysis patients determines if CD allows reduction in CHD during HD compared with AD. After enrolment, intradialytic heparin is minimised during a two-week run-in period using a standardised protocol based on a visual clotting score. Patients still requiring intradialytic heparin after the run-in period are randomised to two weeks of HD with AD followed by two weeks of CD (Sequence 1) or two weeks of HD with CD followed by two weeks of AD (Sequence 2). The primary outcome is the change in CHD with CD compared with AD. Secondary outcomes include metabolic and haemodynamic parameters, and dialysis adequacy.

**Discussion:**

This randomised controlled trial will determine the impact of CD compared with AD on CHD during HD.

**Trial registration:**

ClinicalTrials.gov NCT01466959

## Background

During haemodialysis (HD), blood is exposed to an extracorporeal circuit which activates thrombogenic pathways and clotting [1]. Clotting in the dialysis circuit decreases HD efficiency, and increases nursing workload and costs [2]. Even subclinical clotting of the dialysis circuit may reduce effective dialyser surface area and pore size, decreasing both small and middle solute clearance. Additionally, activation of the coagulation pathway in HD patients may be associated with increased inflammation and accelerated atherosclerosis [3].

In North America, unfractionated heparin is the most common anticoagulant used to prevent clotting during HD [4, 5]. Although effective and inexpensive, it has a narrow therapeutic window without adverse bleeding. Since heparin provides systemic anticoagulation, it is contraindicated in the setting of active bleeding, trauma, pericarditis, intracerebral haemorrhage, thrombocytopenia, coagulopathy, and peri-operative care [6–9]. Furthermore, heparin may lead to osteoporosis, dyslipidaemia, hyperkalaemia, and immune-mediated heparin-induced thrombocytopenia [2, 10–16].

An alternative to systemic heparin anticoagulation for HD is citrate dialysate (CD). CD contains a small amount of citric acid rather than acetic acid as the acidifying agent, at one-fifth the concentration of citric acid used for regional citrate anticoagulation. There are several observational studies [17–27] and two randomised trials using CD in HD patients [28, 29]. These studies demonstrate that CD is associated with decreased clotting of the haemodialysis circuit [17], decreased need for anticoagulation [24, 26, 29], increased dialyser reuse [19], and increased small and middle solute clearance [18–20, 26–28, 30]. These benefits are from an anticoagulation effect that is limited to the extracorporeal circuit without systemic coagulation activation [28]. Other associated benefits of CD include decreased acidosis [18, 22, 28], decreased inflammation [27], decreased oxidative stress [25], improved anaemia [22], improved nutrition [22], and greater haemodynamic stability [21, 28, 31]. CD is well tolerated with no unstable metabolic parameters and no documented adverse effects [18, 22, 29], even in paediatric patients with acute kidney injury [32], in critically ill patients [17], and patients with advanced liver failure [33].

We describe our protocol for a six-week, double-blinded, randomised cross-over trial that investigates whether CD results in reduced cumulative heparin dose (CHD) compared to acetate dialysate (AD) in chronic HD patients. Secondary outcomes address safety concerns, and include metabolic and haemodynamic parameters, and dialysis adequacy.

## Methods

### Study setting

This randomised crossover clinical trial is performed within three community HD units in Calgary, Alberta, Canada, where unfractionated heparin is used for standard intradialytic anticoagulation. All participants provide written informed consent. The study protocol is approved by the Conjoint Health Research Ethics Board at the University of Calgary, and is conducted in accordance with the Declaration of Helsinki (ClinicalTrials.gov, NCT01466959).

### Study population

Eligible patients are ≥ 18 years, and on outpatient conventional HD three times per week for at least three months. Exclusion criteria include: contraindication to heparin, heparin-free HD, known clotting disorder, warfarin therapy, dialysing with a dysfunctional central venous catheter (blood flow rate consistently < 300 mL/min and/or frequent use of thrombolytic), vascular access dysfunction, planned vascular access conversion or procedure during the study period, use of high calcium dialysate, active medical issue, planned kidney transplant during the study period, planned conversion of dialysis modality (peritoneal dialysis, nocturnal dialysis) during the study period, or inability to provide informed consent. Patients with recent modifications to their erythropoiesis-stimulating agent dose are reviewed by the investigator for eligibility.

### Study protocol

**Fig. 1:**
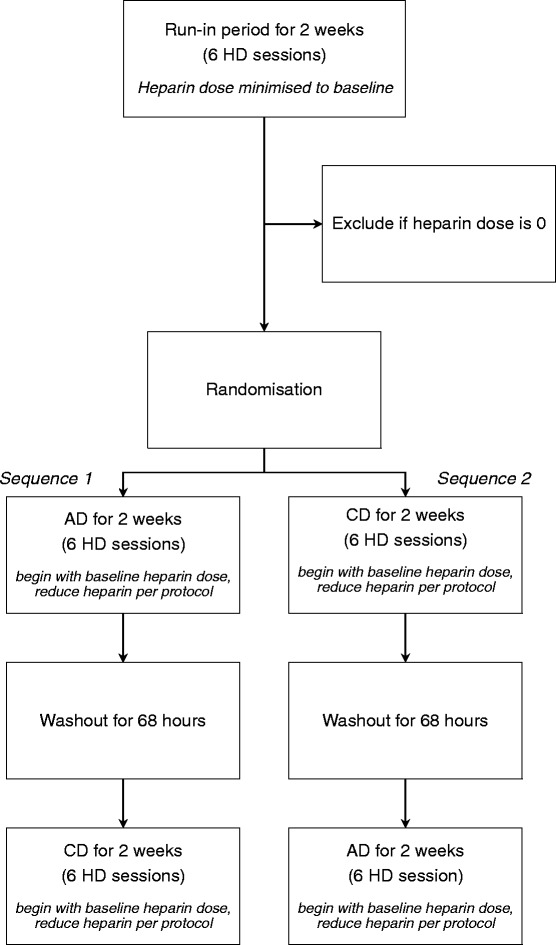
Study Flow Diagram

After enrolment, each patient’s dose of intradialytic heparin is minimised during a two-week run-in period (Fig. 1) using study protocol (Table 1) which is based on a standardised visual clotting score [34]. The visual clotting score is quantified by inspecting the venous chamber and all four quadrants of the dialyser at the end of the HD session (0: clear dialyser/no clots; 1: few strands/small clots; 2: half clotted; 3: three-quarters clotted; 4: completely clotted, unable to return blood) (Table 1). The heparin dose is reduced to reach a target visual clotting score of 2, then increased slightly and maintained, to achieve the lowest possible dose required to prevent clotting (Table 1). The CHD during the last HD session of the run-in period is considered to be the patient’s baseline heparin dose. Patients still requiring intradialytic heparin after the two-week run-in period, and who still meet inclusion and exclusion criteria, are randomised to one of two treatment sequences (Fig. 1). For sequence 1, patients undergo six HD sessions over two weeks with AD (control), followed by six HD sessions over two weeks with CD (intervention). For sequence 2, patients undergo six HD sessions over two weeks with CD (intervention), followed by two weeks of AD (control) (Fig. 1). Patients begin each study phase (AD and CD) with their baseline intradialytic heparin dose, determined at the end of the run-in period. Nurses subsequently adjust intradialytic heparin dose per protocol (Table 1) during each HD session of each study phase. At the end of the first study phase (two weeks), a 68-h washout period ensures no potential for a carry-over effect. After the wash-out period, patients cross-over to the other treatment for the next two weeks beginning again with their baseline heparin dose (Fig. 1). A two-week duration for each strategy is chosen to ensure adequate time to attain the minimum required heparin dose.

**Table 1:** Visual Clotting Score and Heparin Protocol

Clotting Score	Dialyser/Venous Chamber Appearance (at the end of each HD session)	Heparin Dose Adjustment (for the next HD session)
0	Clear	Decrease^a^ boost by 200 units *and* decrease running dose by 200 units/h
1	Few strands/small clot	Decrease^a^ boost by 200 units *and* decrease running dose by 200 units/h
2	½ clotted	Increase boost by 200 units *and* increase running dose by 200 units/h and *maintain* this new heparin dose for the remainder of this study phase only.
3	¾ clotted (able to return blood)	Increase boost by 300 units *and* increase running dose by 300 units/h
4	Completely clotted (unable to return blood)	Increase boost by 400 units *and* increase running dose by 400 units/h

*HD* haemodialysis

NOTE

1) If there is unusually prolonged bleeding from vascular access sites post-HD, for the next HD session, decrease^a^ heparin running dose by 200 units/h and turn heparin off 30 min earlier than usual

2) If there is a discrepancy in score between the dialyser and the venous chamber, the heparin dose adjustment is based on the dialyser clotting score

^a^The minimum possible heparin boost and running dose are 500 units (boost) and 500 units/h. If the heparin dose is to be decreased to less than 500 units (boost and/or running dose) for the next HD session, discontinue the boost and/or the running dose

Randomisation is determined by a computer generated random number list and concealed in sequentially numbered, opaque, sealed envelopes to ensure allocation concealment. Following informed consent and enrolment, the envelope containing allocation sequence is provided to the research coordinator who implements the sequence assignment according to protocol. The intervention and control dialysates, CD and AD, are packaged identically by the manufacturer (Chief Medical Supplies, Ltd., Calgary, Alberta, Canada) for blinding of investigators, patients, and health care staff. All data is confidential, and entered into electronic records by an independent data entry clerk who is blinded to patient allocation.

All study patients are dialysed using Gambro Phoenix® dialysis systems (Gambro-Hospal, Mirandola, Italy) at least three times per week. Each patient’s target weight and dialysis prescription are optimised at the study start by the rounding nephrologist using clinical assessment. Standard dialysis prescriptions (blood flow rate 300–450 mL/min, dialysate flow rate 500 mL/min, dialysate temperature 0.5 °C less than patient temperature, dialysate sodium 137 mmol/L) are maintained unless otherwise prescribed. Dialysers are high-flux, polysulfone (REXEED™, Asahi Kasei Medical Co. Ltd., Tokyo, Japan). HD prescriptions, target weights, and antihypertensive medications are not changed during the study period. The intervention dialysate, CD, contains 2.4 mEq/L of citric acid (Citrasate®, Advanced Renal Technologies, Bellevue, WA), and the control dialysate, AD, contains 4 mmol/L of acetic acid.

### Study endpoints

Primary outcome measures are the absolute and percent change in CHD from baseline with CD compared with AD. CHD is defined as the total heparin received per dialysis session. Secondary outcomes are the effect of CD compared with AD on systemic anticoagulation (prothrombin time [PT]/international normalised ratio [INR], activated partial thromboplastin time [aPTT]), intradialytic metabolic parameters (serum total serum calcium, ionized calcium, magnesium, bicarbonate), anaemia, haemodynamic stability (blood pressure, heart rate, corrected QT interval, number of HD sessions complicated by intradialytic hypotension), dialysis adequacy (single pool Kt/V, urea reduction ratio, beta-2-microglobulin [B2M]), systematic inflammation (C-reactive protein [CRP]), bleeding time after HD, and bleeding events. Also, the visual clotting score and the dialyser fibre bundle volume (FBV) (as measured by the ultrasound dilution technique [35]) are correlated. Lastly, the intra-rater and inter-rater reliability of the visual clotting score is assessed.

## Data collection

### Baseline characteristics

Baseline patient demographics (age, gender, HD vintage, race), comorbidities, cause of ESRD, labs, and HD prescription (dialyser, dialysate composition and temperature, blood and dialysate flow rates, target weight, anticoagulation) are extracted from HD charts and the Southern Alberta Renal Program database [36].

### Cumulative heparin dose

The CHD is calculated per HD session, and determined from HD run sheets. Specifically, CHD is the initial heparin bolus, plus the hourly heparin infusion rate multiplied by the time on HD during which heparin is infused, per session, in heparin units. For the primary outcome, the CHD for the last two HD runs of each study phase are recorded, averaged, and used to calculate change from baseline CHD. The last two HD sessions of each phase is chosen to allow adequate time in each phase for heparin dose minimisation.

### Visual clotting score, inter-and intra-rater reliability

The clotting score is a visual grade of dialyser and venous chamber clotting (Table 1). To ensure consistency, scoring of dialyser/venous chamber clotting for heparin dose adjustments are performed at the end of each HD session by a single, trained research nurse. A second HD nurse independently and blindly scores the dialyser/venous chamber appearance; this second clotting score is recorded to assess inter-rater reliability, but is not used to adjust the heparin dose per protocol. To assess for intra-rater reliability, at the end of each HD session during the run-in phase, the dialyser quadrants are captured in four photographs by the same trained research nurse, using a digital camera on a tripod, with standardised lighting, at a standardised distance and height from the dialyser. The dialyser is rotated by 90° for each of the four photographs, to visually capture the full circumferential view of the dialyser. The photographs are re-presented in a blinded fashion to the same research nurse at a later date to be re-scored.

### Metabolic, anticoagulation, and inflammatory parameters

Serum total calcium, ionized calcium, albumin, magnesium, bicarbonate, haemoglobin, urea, PT/INR, aPTT, B2M, and CRP are collected at the beginning of the first HD session of the study (whether randomised to Sequence 1 or 2), and drawn again both pre and post HD for the last HD sessions of each study phase. Ionized calcium is drawn pre and post HD during the first and last HD session in each study phase.

### Haemodynamic parameters

Blood pressure and heart rate are measured and recorded by HD nurses pre and post HD, and every 30 min during HD. The number of HD sessions complicated by intradialytic hypotension (IDH) during each study phase is recorded. IDH is defined as a drop in the systolic blood pressure (SBP) by ≥ 20 mmHg to < 100 mmHg with patient symptoms or nursing/physician intervention. If the patient’s pre-HD SBP is < 100 mmHg, then IDH is defined as a drop in SBP ≥ 10 mmHg with patient symptoms or nursing/physician intervention. An electrocardiogram is performed at baseline (after the run-in phase), as well as pre and post HD during the last HD session in each two week study phase.

### Dialysis adequacy

Single pool Kt/V is determined for each HD session using Diascan® (Hospal-Gambro, Mirandola, Italy), which measures the dialysance of sodium through the dialyser membrane corrected for recirculation and ultrafiltration [37], and which is a validated method for measuring urea clearance [38]. In addition, the urea reduction ratio is calculated for the last HD session of each study phase using the equation ((urea pre—urea post)/urea pre).

### Bleeding time and bleeding events

For the last two HD sessions of each study phase, the bleeding time post-HD is recorded and averaged in patients who have a fistula or graft as their vascular access. Bleeding events are documented and classified into: 1) major bleeding (fatal bleeding, overt bleeding associated with a haemoglobin drop ≥ 20 g/L and/or transfusion with 2 units of red blood cells, retroperitoneal/intracranial/intraspinal/intra-ocular/pericardial/non-traumatic intra-articular bleeding), 2) clinically important non-major bleeding (clinically overt bleeding requiring hospital admission or visit to a medical facility or leading to intervention), and 3) minor bleeding (any bleeding episode not meeting criteria for the preceding bleeding categories).

### Fibre bundle volume

FBV is the space within the blood compartment of hollow fibre dialysers [35]. Using the ultrasound dilution technique (Transonic® HD02 monitor, Transonic Systems Inc.®, Ithaca, NY, USA), dialyser FBV is measured twice (and the mean recorded) during both the first and last 30 min of the mid-week HD sessions throughout the entire study period (including the run-in phase). The change in FBV during HD represents the quantity of clot in the dialyser [35] and is determined using the equation ((FBV pre—FBV post)/FBV pre).

## Sample size calculations

Previous studies have demonstrated that CHD is reduced by 30 % with the use of CD [39]. Locally in Calgary, the mean CHD is 3000 units (standard deviation 900 units) per HD session in 85 % of HD patients. Based on this, and taking into account a 30 % dropout rate, we estimate that 20 patients are required to demonstrate a 30 % (1000 units) reduction in CHD with CD compared to AD (alpha 0.05, power 80 %). We use the method of Frison and Pocock for the ANCOVA model, and Rabe-Hesketh and Skrondal for the mixed linear model solution (StataCorp LP version SE 11.0, College Station, TX, USA).

## Statistical analysis

Baseline characteristics will be presented as means and 95 % confidence intervals (CIs) or medians and interquartile range (IQR) for continuous variables, and absolute and relative proportions for categorical variables. To determine the effect of CD compared with AD on reduction in CHD (the primary outcome) a random intercept linear mixed model will be used, with CD or AD as the main exposure, subjects as source of random effects, baseline heparin dose values as covariate (at least 2 of 3 measures from the first of two weeks of each period), and final heparin dose values as outcome (at least 2 of 3 measures of the second week of each period). This model takes into account the correlation of data caused by repeated measures for each subject given the crossover design of the study. Treatment, treatment period, and treatment sequence will be modelled as fixed effects. If there is no evidence of period or carry-over effects, all comparisons will be presented relative to AD, without the need for consideration of carry-over effects.

## Discussion

CD has the potential to decrease intradialytic anticoagulation requirements, and improve patient outcomes. However, existing literature comparing CD to AD is limited. Most studies are small, observational in nature, and focus on dialysis adequacy, dialyser reuse/clotting, or metabolic/inflammatory/haemodynamic parameters [17–23, 25, 27]. Only two prospective randomised studies comparing CD to AD have been performed. One single-centre, unblinded crossover study randomised twenty-three maintenance HD patients to first receive CD without anticoagulation or AD with a Tinzaparin bolus [29]. If the CD session revealed early clotting of the venous chamber or dialyser, a Tinzaparin bolus at half the usual dose could be given, and repeated once. During the HD sessions with CD, 50 % of patients did not require anticoagulation, 32 % required a reduced Tinzaparin bolus, and 18 % stopped HD early due to clotting without Tinzaparin. Of those who received anticoagulation with CD, the median dose of Tinzaparin received was 40 % of the standard dose. There was no difference in dialysis adequacy between groups [29]. The main limitation of this study is that the two groups were not treated equally; unlike the CD group, no attempt was made to reduce anticoagulation in the AD group [29]. Another single-centre, single-blinded, crossover study randomised twenty-five clinically stable maintenance HD patients to receive either AD or CD weekly in an alternating fashion for four weeks [28]. The primary outcome was systemic haemodynamics; secondary outcomes were coagulation activation, acid–base balance, calcium balance, and dialysis adequacy. Compared with AD, the CD group had significantly lower blood pressure and peripheral vascular resistance without any difference in stroke volume. However, the decrease in SBP was ablated with dialysate calcium supplementation in the last study week. CD resulted in a significant increase in pre-HD bicarbonate levels, lower post-HD ionized calcium, and higher Kt/V without any effect on coagulation parameters [28]. This study did not assess the effect of CD on intradialytic heparin dose (heparin was used per usual protocol in both groups); the effect of CD on IDH was also not examined [28].

Four observational studies specifically address heparin use with CD in maintenance HD patients [24, 26, 30, 39]. One study included 277 chronic HD patients from eight HD units in an eight-week long, open-label, sequential four-phase study [26]. The primary outcome was change in mean effective conductivity clearance with CD (and reduced heparin) versus AD (and standard heparin dose). Each study phase was two weeks long (6 HD sessions). In the first baseline phase, patients received AD and 100 % of their standard heparin dose. In subsequent phases, patients received CD and decreasing amounts of heparin (second phase: CD and 100 % of standard heparin dose; third phase: CD and 80 % of standard heparin dose; fourth phase: CD and 2/3 of standard heparin dose). At the end of the study, there was no difference in dialysis adequacy between baseline (AD with 100 % heparin) and subsequent phases (CD with decreased heparin). Even with a 33 % reduction in heparin, there was no difference in HD circuit clotting, and dialysis adequacy was maintained [26]. This study did not assess if a reduced heparin dose was possible with AD [26]. In another prospective study, chlorhydric-acid based dialysate was compared with CD in ten patients treated with post-dilution online haemodiafiltration [24]. During treatments with CD, the heparin dose was decreased by half, then completely discontinued. In 120 CD sessions without heparin, dialysis adequacy was unchanged, and only one clotting episode related to vascular access stenosis was experienced [24]. Two further observational studies, published in abstract form only, included 20 and 31 chronic HD patients, respectively, who had prolonged bleeding from vascular access needling sites post HD. Patients were switched from AD to CD, and intradialytic heparin doses were reduced by 30 % [19, 39] after two months, and 55 % [30] after four months, respectively. In both studies, heparin reduction with CD was achieved without decrease in dialysis adequacy [30, 39].

Although existing literature suggests that CD use during chronic HD allows intradialytic heparin reduction without sacrifice of dialysis dose, and without other adverse effects, this has yet to be investigated in a rigorous trial. We have presented in detail the motivation, design and strategy for our randomised controlled trial, including methods of recruitment, randomisation, allocation concealment, dialysis intervention, outcome assessment, and data collection. The study protocol is developed according to Standard Protocol Items: Recommendations for Interventional Trials (SPIRIT) 2013 [40]; results will be reported following the Consolidated Standards of Reporting Trials (CONSORT) statement [41].

In summary, our randomised crossover trial of chronic HD patients will determine if CD decreases intradialytic heparin requirements, and its affect on haemodynamic stability and dialysis adequacy. Our study will answer a clinically relevant and important question which has not previously been addressed by a well-designed randomised trial.

## Abbreviations

HD: Haemodialysis
CD: Citrate dialysate
CHD: Cumulative heparin dose
AD: Acetate dialysate
PT: Prothrombin time
INR: International normalised ratio
aPTT: Activated partial thromboplastin time
B2M: Beta-2-microglobulin
CRP: C-reactive protein
FBV: Fibre bundle volume
IDH: Intradialytic hypotension
SBP: Systolic blood pressure
CIs: Confidence intervals
IQR: Interquartile range
